# Cohort Profile: the Office for National Statistics Longitudinal Study (The LS)

**DOI:** 10.1093/ije/dyy243

**Published:** 2018-12-12

**Authors:** Nicola Shelton, Chris E Marshall, Rachel Stuchbury, Emily Grundy, Adam Dennett, Jo Tomlinson, Oliver Duke-Williams, ONS Staff, Wei Xun

**Affiliations:** 1 Centre for Longitudinal Study Information and User Support (CeLSIUS), Department of Epidemiology and Public Health, University College London, London, UK; 2 Institute for Social and Economic Research, University of Essex, Colchester, UK; 3 Centre for Advanced Spatial Analysis; 4 Department of Information Studies, University College London, London, UK; 5 Longitudinal Study Development Team, Office for National Statistics, Titchfield, UK

## Why was the cohort set up?

Two factors were particularly important in the decision to set up the [Office for Population Censuses and Surveys (OPCS), now Office for National Statistics (ONS)] Longitudinal Study (the LS) in 1974.^1^ These were concern over the limitations of the occupational data collected at death registration which were used to calculate occupational mortality rates, and a need for more information on fertility patterns, particularly changes in birth spacing. It was recognized that existing data sources were inadequate for analysis of mortality differentials, particularly by occupation, due to bias resulting from the fact that denominator (population) data came from the Census and numerator (deaths) data from vital registration. Employment profiles that reflected lifetime experiences rather than last job from the death certificate were required for detailed occupational mortality analyses. More information on birth spacing and social and family influences on fertility patterns was also needed, the early 1970s representing the ‘birth dearth’ period when policy makers were very concerned about fertility patterns. The then Office for Population Censuses and Surveys (OPCS) had developed considerable experience in record linkage studies following up particular occupation groups (and members of specific studies), a process facilitated by development of the National Health Service Central Register (NHSCR). In the early 1970s, OPCS decided to make better use of existing resources by establishing a longitudinal study based on linked census and vital registration data (births, deaths, cancer registrations). The usefulness of the LS for migration and sociodemographic studies was also anticipated.

The initial sample was drawn from the 1971 Census on the basis of birthday, in order to facilitate linkage. All those born on four undisclosed birthdays per year were included, giving a sample amounting to just over 1% of the population of England and Wales. The study has been maintained as a continuous multi-cohort through the addition of new births and immigrants with the same birth date, and includes individual-level data from five Censuses (1971, 1981, 1991, 2001, 2011) as well as linked information on births, deaths and cancer registrations. Access to anonymized data for research purposes is permitted under strict access conditions which include useage of microdata in an ONS secure data laboratory.

The LS is representative of the whole population of England and Wales, including those in non-private households and all age groups; and it includes census information about other people in sample members’ households at each census, which provides additional opportunities for examining intergenerational continuities and changes. The ‘width’ of the sample in terms of size means it is possible to study relatively small groups, such as members of particular ethnic minority groups or older people resident in institutional settings (a group excluded altogether from most surveys). The ‘depth’ of the study over time makes it increasingly valuable for research including a life course or intergenerational perspective. A further strong advantage of the LS is minimal bias due to non-response or attrition, as census coverage is good and rates of linkage high.

## Who is in the cohort?

This is a 1% dynamic sample of all persons of any age or gender, identified as having an LS date of birth (one of four dates, spread through the year) and usually resident in England and Wales, who completed a census form and have joined through birth or immigration, since 1971 (Tables 1–5).

**Table 1.** LS data collection and sample sizes

Phase	Measurements	Sample size *n* (to nearest 1000)
Baseline 1971	Self-reported employment, education, marital status, area, housing tenure and characteristics, household composition (see Table 5 for full details)	524 000
Follow-up^a^ 1981	As 1971	533 000
Follow-up^a^ 1991	As 1971 plus limiting long-term illness and ethnicity	537 000
Follow-up^a^ 2001	As 1991 plus general health, religion and care-giving	538 000
Follow-up^a^ 2011	As 2001 plus language spoken, national identity and passport(s) held	581 000
Ongoing	The LS database is updated every year with event data from a number of sources, including: deaths of LS members (up to eight causes of death [up to 16 from 2014]); LS members being widowed; female LS members giving birth; and LS members being diagnosed with cancer	

Source: ONS LS.

^a^The baseline sample will be followed up, but as the LS is a dynamic sample, the subsequent ‘follow-up’ sample also includes new members at each census.

**Table 2.** Numbers of LS members: life events from record linkage until 2015

	Time period					Total
	1971–81	1981–91	1991–2001	2001–11	2011–15	
Mortality	61 070	64 910	64 900	61 270	28 930	281 060
Widow(er)hoods	22 300	22 100	22 150	19 810	9390	95 750
Live births to sample mothers	64 760	68 560	70 350	74 070	37 790	315 520
Stillbirths to sample mothers	570	360	360	380	140	1810
Infant mortality to sample mothers	780	610	500	440	110^a^	2420
Cancer registrations	19 690	27 920	37 740	47 880	27 490^b^	160 730

Source: ONS LS.

Totals may not sum due to rounding.

^a^None processed since the end of 2013.

^b^Includes a small number of 2016 cancer registrations.

**Table 3.** Numbers of LS members: self-reported health status from census

		1991 *n* (%)	2001 *n* (%)	2011 *n* (%)
Limiting long-term illness (LLTI)				
Has LLTI				
Limited a lot (2011)		71 880 (13)	100 350 (19)	50 850 (9)
Limited a little (2011)				56 100 (10)
No LLTI		472 000 (87)	437 850 (81)	476 690 (82)
Total		543 880	538 200	583 640

Self-rated health (SRH)				
Very good		Not reported		270 520 (46)
Good			365 300 (68)	200 440 (34)
Fairly good			122 030 (23)	
Fair				78 930 (14)
Not good			50 880 (9)	
Bad				26 210 (4)
Very bad				7560 (1)
Total			538 210	583 650

Source: CeLSIUS analysis; Data ONS LS.

Totals may not sum due to rounding.

**Table 4.** Number of deaths 2002–10 and death rates by age group at death

	Men		Women	
Age group at death	Deaths *n*	Death rate(*n*/pyr)*1000	Deaths *n*	Death rate(*n*/pyr)*1000
65–74	4448	21.60	3218	14.30
75–84	7833	64.80	7557	45.20
85–94	5209	173.60	9128	139.20
95+	602	404.10	2237	344.60
Total	18 092		22 140	

Source: CeLSIUS analysis. Data ONS LS.

pyr, per year.

**Table 5.** Number and distribution (%) of deaths, by 2001 characteristics and place of death, among persons aged 65 and over in 2001, who died between the 2001 and 2011 censuses

		Men		Women	
Characteristics at 2001 (except place of death)		Deaths *n*	%	Deaths *n*	%
Type of household	Private household	16 820	94.6	19 970	87.3
	Communal establishment	960	5.4	2890	12.7
Housing tenure	Owner	11 790	66.3	12 510	54.7
	Renter	4480	25.2	6340	27.7
	Other	1510	8.5	4020	17.6
Marital status	Single	1330	7.5	1690	7.4
	Married	11 230	63.2	6310	27.6
	Divorced/separated	1020	5.7	980	4.3
	Widowed	4190	23.6	13 890	60.7
Has longstanding illness	Yes	11 240	63.2	16 010	70.0
	No	6540	36.8	6860	30.0
Self-rated health	Good	4570	25.7	5060	22.1
	Fair/poor	13 210	74.3	17 810	77.9
Place of death	Hospital/hospice	6790	38.2	7710	33.7
	Care/nursing home	2510	14.1	6080	26.6
	Other	8480	47.7	9080	39.7
	Total	17 770	100.0	22 870	100.0

Source: ONS LS.

Totals may not sum due to rounding.

## How often have they been followed up?

From the 1971 Census onwards, the LS has been maintained in the following manner.

1. Deaths of LS members are recorded but all preceding data are retained.
2. Any child subsequently born in England and Wales on one of the four LS birth dates is entered into the study.
3. Any immigrant registering with the NHS and declaring their date of birth as one of the LS birth dates is entered into the study.
4. Anyone identified at subsequent censuses as having an LS date of birth and not already in the LS, is entered into the study. Existing LS members have the new census data added to their records.
5. There are no other routes of entry to the study.
6. Any emigrant declaring their date of birth as one of the LS birth dates has their embarkation date recorded but is retained in the study.

## Life events are also linked to LS members, as follows.

1. Deaths are added annually: the death record includes the underlying cause of death and up to eight contributory causes (and up to 16 causes from 2014). These are coded to ICD8 (1971–81), ICD9 (1981–2000) or ICD10 (2000 onwards). In addition, other attributes of the deceased are also held, e.g. ‘Type of establishment’ if they died in a communal establishment.
2. Births and stillbirths registered to mothers who are LS members are added annually; this includes the date of the birth and the babies’ weight (though there are a lot of missing data especially before 1981), gender, place of birth and several other parameters. Births to male LS members were also recorded during the period 1971–81, but this was discontinued because of poor linkage rates.
3. Infant deaths: registered deaths of children born to LS sample mothers are added. Only babies under 1 year of age were recorded up to 1993. Deaths of children under the age 16 years, born from 1993 onwards, are also recorded annually.
4. Embarkations: LS members leaving the country are recorded, as notified to the NHS.
5. Cancer registrations as notified to the cancer registry, using the ICD code current at the time to record the type and site of the growth, are added.
6. Widowhoods and widowerhoods are identified from death registrations (i.e. when the LS member loses their legal spouse, or more recently their civil partner).
7. Entry into armed forces (until 2012) is noted.
8. Entry into long-term psychiatric hospitals (until 1983) is noted.
9. Re-entrants include: LS members (as identified by the NHS) who have emigrated but then returned; left the armed forces; or left a long term psychiatric hospital.

The LS life events tables are generally updated once per year. Late notifications (e.g. of deaths abroad) mean that counts for some years already available will increase with each update. The data on LS members are enhanced (no data are ever deleted) by the addition of new data at 10-yearly intervals as information from the decennial censuses becomes available. The total number of LS members following the 2011 Census is now more than one million—this includes those who have died in the intervening period (5000–8000 per year since 1971, offset by a roughly similar number of births). The number present at any one time point has risen slightly with each census, but ranges between 524 000 and 581 000. The LS is maintained and updated by ONS and makes secondary use of data collected for other purposes. Consent is not required as this work is carried out as part of ONS’s statutory functions as laid out in the Statistics and Registration Service Act 2007.

## What has been measured?

The census gathers a large amount of sociodemographic data every 10 years. The data available consist of the responses to the census questions and some other variables (e.g. social class) derived from relevant census variables. The 1971 Census asked ever-married women then aged 16–59 how many children they had and the year of the children’s births, giving a baseline idea of fertility. The 1971 Census asked for address 5 years ago, 1 year ago and the present address. Subsequent censuses only asked for current address and address 1 year ago. Migration studies therefore have 11 possible residential locations for people identified at 1971, alive in 1966 and still alive in 2011. Additionally, 10-year migration indicators have been derived by comparing address, or postcode district, of members in successive censuses. In the early years of the LS (1971–74), data from moves between Family Practice areas were also recorded. Table 6 shows key variables included in the 1971 and subsequent censuses.

**Table 6.** Topics available in the ONS LS by time period

			1971	1981	1991	2001	2011	Annually from 1971
Person (each person in household or communal establishment)								
	Sex		√	√	√	√	√	√
	Year of birth		√	√	√	√	√	√
	Relationship to head of household/household reference person		√	√	√	√	√	
	Marital status (inc. civil partnerships)		√	√	√	√	√	
	Number of live-born, legitimate children (women under 60 only)		√					
	Marital history (women under 60 only)		√					
	Usual resident or visitor at address of enumeration		√	√	√	√	√	
	Geographical location of second address, and reason for going there						√	
	Country of birth		√	√	√	√	√	√
	Country of birth of mother and father^a^		√					√
	National identity (self-chosen)						√	
	Passports held						√	
	When arrived in UK (if born elsewhere)						√	
	Length of intended stay in UK (if born elsewhere)						√	
	Geographical location 1 year previously		√	√	√	√	√	
	Geographical location 5 years previously		√					
	Ethnic group				√	√	√	
	Religion (response is voluntary)					√	√	
	Welsh language (Wales only)		√	√	√	√	√	
	Main language; facility in spoken English						√	
	Qualifications (question varies over time)		√	√	√	√	√	
	Whether working/unemployed/retired/inactive last week		√	√	√	√	√	
	Whether student last week		√	√	√	√	√	
	Employment status (full/part-time, self-employed, apprentice etc.)		√	√	√	√	√	
	Industry		√	√	√	√	√	
	Occupation		√	√	√	√	√	√
	Occupation 1 year previously		√					
	Year last worked					√	√	
	Hours of work		√	√	√	√	√	
	Geographical location of workplace		√		√	√	√	
	Journey to work		√		√	√	√	
	Whether has limiting long-term illness				√	√	√	
	Health in past year					√	√	
	Whether informal carer; hours per week					√	√	

Household								
	Geographical location (at various levels)		√	√	√	√	√	√
	Nature of accommodation (house/flat/caravan etc.)			√	√	√	√	
	Housing tenure		√	√	√	√	√	
	Whether accommodation is self-contained		√	√	√	√	√	
	Number of rooms		√	√	√	√	√	
	Cars/vans available		√	√	√	√	√	
	Amenities (cooking, hygiene, heating etc.)		√	√	√	√	√	
	Communal establishment: type, and number of rooms		√	√	√	√	√	

Life events								
	Date and place of own birth (1971 onward)							√
	Birthweight (incomplete before about 1983)							√
	Whether multiple birth; type and whether siblings were live-born							√
	Date and place of own death							√
	Cause of death							√
	Death of legal spouse (i.e. widowhood/widowerhood)							√
	Birth of child(ren): date and place (women only)							√
	Birthweight of child(ren) (women only; incomplete before 1983)							√
	Whether child(ren) live or stillborn							√
	Death of child (women only; death of infant only until 1993)							√
	Birthweight of deceased child							√
	Cause of death of deceased child							√

Source: ONS LS.

^a^(i) Question asked at 1971 Census; (ii) information collected from parents at registration of birth.

## What has it found? Key findings and publications

The LS has provided evidence with academic and non-academic impact for social policy issues such as:

- inequalities in health, employment, education and geography;
- equal opportunities for women, ethnic groups and the long-term sick;
- social exclusion, including long-term outcomes of education and employment status;
- economic integration of migrant groups;
- housing and geographical mobility;
- family policy, including early/late parenthood, different childbearing patterns of advantaged and less advantaged groups, and cohabitation.

The LS has been used to provide unique information to support a series of major reports for government on health and mortality: *Inequalities in Health*, 1980 (the Black Report)^2^; *The Health Divide: Inequalities in Health in the 1980s*, 1987 (the Whitehead Report);^3^*Independent Inquiry Into Inequalities in Health Report*, 1998 (the Acheson Report);^4^ and the *Strategic Review of Health Inequalities in England Post-2010: Fair Society, Healthy Lives* (the Marmot Review).^5^

The LS has also been used for analysis of work on pensions. The first report, *Pensions: Challenges and Choices*, in 2004,^6^ was followed in 2005 by the Turner Report: *A New Pension Settlement for the Twenty-first Century.*^7^ Both reports include information on trends in life expectancy at 65, by social class. Subsequently research from the LS has fed into the state pension age review in 2017.^8^

The Dilnot Report: *Fairer Care Funding* was published in 2011.^9^ The size of the population in long-term residential and nursing home care at any one point in time depends on rates of admission and length of stay. The submission used data from the LS on the survival of older people who in the 2001 Census were recorded as residents of residential care homes, nursing homes or other types of communal establishment, and examined differentials in the survival of this population by characteristics including: broad type of establishment (residential, nursing or other); gender; and marital status in 2001. It also used information on place of death, to assess the assumption that residents in communal establishments of various types in 2001 remained in institutional care throughout the follow-up period (from the 2001 Census to the end of 2008).^10^

Social mobility continues to be of significant political concern; a report for the Joseph Rowntree Foundation was published in 2005, which traced patterns of intergenerational social mobility for children born between the late 1950s and mid-1970s from different ethnic groups in England and Wales. Key findings included: the children of parents in higher social classes were more likely to end up in higher social classes themselves; and most minority ethnic groups showed high levels of children moving into a higher class than their parents. The stability of couple partnerships is also of interest to policy makers. The paper: ‘Do partnerships last?, comparing marriage and cohabitation using longitudinal census data’ was published in 2010.^11^ The research used a sample of adults who were in a partnership (married or cohabiting) in the 1991 Census of England and Wales, and then explored whether these individuals were living with the same partner in 2001. Main findings include: 82% of married adults aged between 16 and 54 in 1991 were still living with the same partner in 2001, compared with 61% of cohabiting adults; adults were less likely to remain with the same partner if, in 1991, they were younger, had no dependent children living in the household, had a limiting long-term illness, had previous experience of partnership dissolution, had no higher qualifications or were unemployed. This paper now is cited in the A-level Sociology syllabus.

Academic impact is a key feature of LS research. There are many highly cited papers, especially within epidemiology and the social sciences. Examples include sex differences in developmental reading disability,^12^ selective migration and health^13^ and limiting long-term illness and mortality among non-migrant people,^14^ fertility history and health in later life,^15^ socioeconomic status and ischaemic heart disease mortality,^16^ sociodemographic variations in moves to institutional care,^17^ living arrangements and place of death,^18^ accumulated labour market disadvantage and limiting long-term illness,^19^ population change and migration,^20^ and cancer and proximity to power lines.^21^

Recent work drawing significant media attention includes trends in life expectancy at birth and at age 65 by socioeconomic position based on the *National Statistics Socioeconomic Classification, England and Wales*: 1982–86 to 2007–11, produced by ONS. Headline results that the most advantaged men were living longer than the least advantaged women for the first time were published in many national newspapers.^22–25^ A paper on impacts of *in utero* exposure to air pollution using LS data was featured in the *Telegraph*^26–27^ and a paper on chronic health effects of air pollution was widely featured in the press.^28^ A full set of publications is available here at the Census & Administrative Data Longitudinal Studies Hub.^29^

## Main strengths and weaknesses

The strength of the LS is its large sample size (total *N* > 1 000 000), the length of follow-up available (40 years, 1971–2011 for main census data) with life events for LS members available until about 2 years before the current year of analysis. This is by far the largest nationally representative longitudinal dataset in the UK; it allows analysis of small areas (well below local authority level), particular ethnic groups and specific occupational groups. These are not possible with any other longitudinal dataset because of insufficient numbers. In addition to information on LS members, there is information on all persons in their household at any time point. This means that information missing, for example the social class of a child, can be recovered by looking at the social class of their parents. With the long period of follow-up, survival analysis can be performed looking at differences between subjects with far more parameters than just age at death and sex: industry, social class, education and location are all variables that could be entered into the analysis.

Geography (where people live) is consistent at all time points to the geographical identity in England and Wales in 1974. However, researchers will need guidance as to which variables to use as other geographies are in force in the LS in 1991, 2001 and 2011. The lowest geography at which a researcher may generally report results is Local Authority, of which there are just under 350 in England and Wales. Lower level geographies are available for attaching the researcher’s own external data, but the small area geographical variables are removed before the dataset is made available to the researcher. Unusually, the data include persons in communal establishments, so groups such as students and older adults are represented.

Since the LS comprises all persons born on 4 days of the year, the sampling fraction is approximately 1.1% and sampling bias is almost nil. The high tracing rates contribute to the high linkage rate of LS members from census to census (88% 2001 to 2011).^30^ Response rates to the 2011 Census were very high relative to other national censuses, sample surveys and cohort and panel studies, at 94%.^31^ There are changes in study population over time, but this offers the opportunity to look at both a closed cohort and a representative sample of the national population. Table 7 shows the tracing rates for each of the five censuses included in the LS.

**Table 7.** LS tracing rates

Census	Total enumerated	Total traced by NHS Digital^a^	Percent traced by NHS Digital	Forward linkage rates (%)
1971	529 900	523 800	98.8	91.3
1981	536 100	532 600	99.3	90.1
1991	543 900	537 200	98.8	88.0
2001	540 100	537 600	99.6	87.7
2011	585 800	580 600	99.1	N/A

Source: ONS LS.

^a^The same business area located in Southport, Lancashire has always carried out the tracing activity that supports the linkage of LS data. Between 1974 and 2008, this business area was part of OPCS/ONS. When the Statistics & Registration Service Act 2007 was enacted in 2008, this business area moved to the National Information Centre for Health & Social Care (NHSIC). NHSIC became NHS Digital on 1 August 2016.

Comparative analyses of UK data are also possible using e-Datashield for the periods 1991, 2001 and 2011.^32^ Sister cohorts exist for Northern Ireland^33^ and Scotland^34^ and these can be analysed indirectly in any of the three Research Support Units in London, Edinburgh or Belfast, drawing on the strengths of the support teams in all three units and the e-DataSHIELD software. A considerable amount of meta-data are available for the LS, including a data dictionary with sample sizes and variable similarity scores over time.^35^

Relative to cohort and panel studies there is a limited set of questions asked, and there are changes in definitions and questions asked for several variables over time. The main weakness of the LS is the lack of behavioural data. Also the census is every 10 years, so updates are limited, but there are some questions that offer retrospective information such as year left last job and address 1 year ago. As the data are anonymized, but LS members do not know they are part of the study, extreme care has to be taken when reporting results: no cell count less than 10 may be published unless the researcher can demonstrate that a lower cell count is not disclosive and that it is vital to the findings of the research project, but the onus is on the researcher to prove this.

## Can I get hold of the data? Where can I find out more?

The LS data are available to anyone in the UK who can fulfil the requirements of ONS’s Approved Researcher Scheme.^36^ The data can be accessed through the Secure Research Service (SRS) safe setting rooms at ONS offices in London (Pimlico), Hampshire (Titchfield) and South Wales (Newport), or remotely by sending syntax to user support officers to run, and receiving output by return. The Centre for Longitudinal Study Information and User Support (CeLSIUS) provides support for UK-based researchers from the academic, public and third sectors. The LS Development Team at ONS provides support for all other researchers.

The application process is fully detailed on the CeLSIUS website at [www.ucl.ac.uk/celsius] where all the necessary forms can be found under the ‘Using the ONS Longitudinal Study’ section. Significant user support is provided by CeLSIUS and ONS. A synthetic training dataset with a limited range of variables and transitions from 2001–11 is freely available to download under Open Government Licence for testing syntax and sample size estimations.^37^^,^^38^ Synthpop, the process for offering individual synthetic datasets to order, to be accessible on desktops, is under development. Further information can be found at [www.ucl.ac.uk/celsius] and in the *Routledge Handbook of Census Resources.*^31^

CeLSIUS is supported by the Economic and Social Research Council (Award Ref: ES/R00823X/1) and therefore their service is free to academic and public sector researchers in most circumstances. This work contains statistical data from ONS which is Crown Copyright. The use of the ONS statistical data in this work does not imply the endorsement of the ONS in relation to the interpretation or analysis of the statistical data. This work uses research datasets which may not exactly reproduce National Statistics aggregates.

**Conflict of interest:** None declared.

**Profile in a nutshell**

- The Office for National Statistics Longitudinal Study (The LS) was set up to improve social statistics. An approximate 1% sample of the population was obtained from the 1971 Census for England and Wales by selecting anyone of any age born on one of four birthdates.
- The sample has been continuously refreshed by adding new births and immigrants with LS birth dates.
- Census data from 1981-2011 have been linked and planning is underway to link a 6^th^ tranche from the 2021 Census. Data from the 2011 Census was linked for more than 580,000 study members.
- Data is socio-economic and demographic data with self-reported health measurements since 1991 and linkage to mortality and cancer registration from 1971.
